# Understanding readmission after hip fracture: a mixed methods study protocol

**DOI:** 10.1136/bmjopen-2024-094163

**Published:** 2025-07-15

**Authors:** Emma Sutton, Usama Rahman, Hannah Reilly, Caroline Miller, Tarunya Vedutla, Teresa Melody, Ranaganayaki Gudivada, Annie E Topping

**Affiliations:** 1 Department of Nursing and Midwifery School of Health Sciences College of Medicine and Health, University of Birmingham, Birmingham, UK; 2 School of Nursing, AHP and Midwifery, University Hospitals Birmingham NHS Foundation Trust, Birmingham, UK; 3 Barts Bone and Joint Health, Queen Mary University of London, London, UK; 4 University Hospitals Birmingham NHS Foundation Trust, Birmingham, UK; 5 Institute of Mental Health, University of Nottingham, Nottingham, UK; 6 School of Infection, Inflammation and Immunology, University of Birmingham, Birmingham, UK; 7 Birmingham Community Healthcare NHS Foundation Trust, Birmingham, UK

**Keywords:** PUBLIC HEALTH, Hip, Frail Elderly, SOCIAL MEDICINE

## Abstract

**Abstract:**

**Introduction:**

Around 75 000 people suffer from hip fractures yearly in the United Kingdom (UK) leading to significant mortality and morbidity. Although mortality has dropped from 8% to 5% between 2013 and 2023 after hip fractures, those undergoing surgery for hip fractures have a 30-day readmission rate which has remained stagnant at around 11% over the same decade in the UK.

This study protocol describes a mixed-methods investigation (The ARTHUR Study—avoiding readmission after hip fracture) which aims to understand and offer solutions to prevent avoidable 30-day readmission after hip fracture surgery. The study will focus on two hospitals in acute and community settings in a large urban and ethnically diverse city in the UK.

**Methods and analysis:**

We describe two work packages.

Work Package One (WP1) involves analysis of 5 year’s worth of routinely collected health data provided by PIONEER, a Health Data Research UK data hub in Acute Care for our local population. Work Package Two (WP2) will involve semistructured interviews with patients, carers or family members as well as non-participant observations of hospital processes to understand systems-based issues related to readmissions after hip fracture surgery. Although recruitment may be an issue, our timeline for recruitment reflects this. We also aim to recruit a diverse population, which has often been under-represented in studies into hip fractures and aim to explore relevant interventions which can be widely generalisable.

**Ethics and dissemination:**

This protocol was submitted via IRAS: 330074 and obtained UK NHS REC approval via the West Midlands Coventry and Warwickshire Research Ethics Committee (REC 23/WM/0242) on 25 January 2024. The results of this study will be published in relevant scientific journals and presented at orthopaedic, fragility fracture and geriatric specialty conferences and scientific meetings. A lay summary of the findings will be publicly available on the HRA website.

**Strengths and Limitations of this study**

- Mixed-methods approach: the use of both quantitative (health data analysis) and qualitative (interviews and observations) methods provides a comprehensive understanding of the factors contributing to 30-day readmission, offering a well-rounded perspective for future intervention development.
- Diverse population focus: the study intentionally aims to recruit from a large, urban, ethnically diverse city, which increases the potential generalisability of the findings to a broader population, addressing a gap in previous research on hip fractures.
- Potential recruitment challenges: although the timeline accounts for potential difficulties, recruiting a diverse population can be time-consuming and may still present challenges in achieving the target number of participants.
- No immediate trial of interventions: future study is needed to design and test interventions, which may be capable of reducing avoidable readmission after hip fracture.

## Introduction

 A broken hip ‘hip fracture’ is a serious injury which in the UK leads to over 70 000 people needing surgery followed by rehabilitation.^1^ Mortality for hip fracture in England, Wales and Northern Ireland has fallen from 8.4% in 2013 to 6% in 2025.^2^ Yet, the number of 30-day readmissions remains high, dropping by only 1% in England over the last decade (12.7%–11.7%).^3^ Some areas of England experience higher readmission rates than the national average, for example—in parts of the Midlands—13.4% of people who experience hip fracture are readmitted within 30 days.^3^ Some areas within the Midlands also experience high levels of socioeconomic deprivation,^4^ and health and wealth inequality. Individuals with low socioeconomic status (SES) have a more than 25% higher risk of having a fragility fractures^5^ and are also more likely to experience worse postsurgical outcomes including readmission.^6^ Readmitted patients have an increased risk of death, complications and being discharged to a residence which is not their own home.^7^ Readmission is also expensive, costing an estimated £14.5 million per year based on an average length of stay for each readmission after hip fracture of 8.7 days^7^ and costs per day of hospitalisation between £200 and £400.^8^,^10^ Costs per patient for hip fracture are substantially higher in the Midlands than other regions in England.^10^

Some readmission predictors, such as age, cannot be altered, but others are malleable. The proportion of patient readmissions cited to be potentially avoidable ranges between 5% and 79%^11^; with the underlying causes of readmission relating to wound problems,^12^ infection,^13^ rehabilitation issues^13^ and medicines management (patient adherence, monitoring and prescribing errors).^14^ These avoidable readmissions may be reduced by self-care, individualised discharge planning, regional anaesthesia,^15^ collaborative working, improved management of hip fracture pathway documents,^16^ implementing a preoperative fluid management protocol, reporting how soon community therapy will start after discharge, higher volumes of orthopaedic non-consultant doctors and having plans to reconfigure the service.^17^ Despite individual, societal and economic impact of reducing avoidable readmission for older people, it is not yet known which specific combination of interventions or dose is effective in reducing readmissions which could be avoidable. This study will ask:

1. How is avoidable readmission defined and categorised by caregivers and patients?
2. What are the characteristics of patients with a fractured hip who are admitted and readmitted within 30 days?
3. Which interventions are most capable of reducing avoidable readmission after fragility fracture of the hip in the Midlands area of England whose population experiences health and wealth inequality?

## Methods and analysis

Using mixed methods, this study will:

- Explore how avoidable readmission is understood by caregivers and patients.
- Investigate the characteristics of patients with a fractured hip who are admitted and readmitted within 30 days.
- Describe the patient journey and factors affecting service delivery for hip fracture patients in two National Health Service (NHS) Trusts in the Midlands region of England.

### Work package one: PIONEER

Five years of anonymised routinely collected data will be extracted from PIONEER,^18^ a Health Data Research Hub in Acute Care (https://www.pioneerdatahub.co.uk), relating to admitted fragility hip fracture patients from an acute NHS Trust in the Midlands region of England, (1 January 2019 to 31 December 2023). This data will be retrospectively analysed to identify trends such as age, gender, deprivation, ethnicity and length of in hospital stay. Additionally, we will map number of primary patient admissions and readmissions with hip fracture. We will identify reasons for readmission, and we will analyse ward-based assessments, surgical factors and medical factors which may be associated with readmission. Finally, we will explore data that can be used for health economic evaluation on 30-day readmission. PIONEER data points will be collected from:

- Patients with a hip fracture—a hip fracture refers to a fracture occurring in the area between the edge of the femoral head and 5 cm below the lesser trochanter.^19^
- Patients with a fragility fracture of the hip—a fracture that results from minimal trauma (eg, a fall from standing height) or no identifiable trauma at all.
- Patients equal to and over the age of 65.

PIONEER will provide licensed access to anonymised data. PIONEER will conduct a technical security assessment of the proposed data storage environment to ensure it is secure and meets all the license requirements. Data will be analysed under the direction of a PIONEER statistician who will link primary admission data to readmissions to identify trends and correlations. Associations with categorical variables will be analysed using χ² tests or Fisher’s exact as appropriate; for continuous variables, we will use t-tests if normally distributed or Kruskal-Wallis tests. Multivariable logistic regression will be performed.

### Data sharing agreement

The anonymised participant data, analytical code and a data dictionary defining each field will be available to others through application to PIONEER via the corresponding author.

#### Identification of hip fractures, re-admissions and planned analyses

Incident hip fractures will be identified using OPCS (Office of Population Censuses and Surveys), ICD (International Classification of Diseases) and Snomed CT (Systematized Nomenclature of Medicine Clinical Terms) codes through the combined electronic health records system, which is consistently used across all of the included admitting sites. Readmissions will be identified using the same method and primary readmission diagnoses will be analysed based on these codes. A measure of deprivation will be determined using the patient’s primary address linked to freely available public health local super output area (LSOA) data.

#### Work package two: observational field work and interviews

Non-participant observations (where researchers collect data without actually interacting or participating in the activity being observed) of staff clinical meetings in two NHS Trusts in the Midlands region of England will be used to understand system-based challenges affecting service delivery (ie, when readmission could have been avoided/in what circumstances, referral processes); what counts as an avoidable readmission; how people arrive at a decision/how are processes enacted by staff that may impact on avoidable admission. Clinical meetings such as bed capacity and multidisciplinary team (MTD) meetings have been deliberately selected to observe staff clinical decision-making which will provide insight into systems-based challenges which could affect readmission or readmission risk. Patients will not be observed, and no identifiable patient information will be recorded. All observation activities will be conducted by members of the ARTHUR study team who have received training on ethnographic observations and will be delegated to undertake this task.

An observation guide will be used to structure field notes (online supplemental file 1). A total of approximately 12–20 meetings over a 4-month period will be observed. These data will be analysed concurrently using thematic analysis^20 21^ to develop and review codes and work to the principle of information data saturation^20^ estimated to be reached between 12 and 20 meeting observations.

During Work Package Two (WP2) health and social care staff (in care homes and the NHS) and patients and carers either in hospital or at their discharge destination will be interviewed. Interview data will help to understand what avoidable means and explain why patients who experience a fragility fracture of the hip may be readmitted within 30 days. The eligibility criteria for patient participants are:

- Experience of a hip fracture within the last 6 months.
- Equal to or over the age of 65 at the time of sustaining the primary hip fracture.
- Clinical Frailty Score (CFS) below 7 (either documented in the patient health record or assessed by healthcare staff).
- Have capacity to consent.

Patients will be excluded if they are considered by their healthcare team (gatekeepers) not to be well enough to be approached or if they are experiencing postoperative delirium at the point of recruitment.

To be eligible for the study, health and social care staff and carers must have experience of caring for someone who is equal to or over 65 at the time of their hip fracture.

*Identification of patients and carers:* potential participants for the interview study will be identified by members of the patient’s healthcare team. These gatekeepers are able to identify eligible patients, and/or those who care for them for the interview study. A member of the ARTHUR study team will telephone (or visit) the ‘gatekeeper’ to identify patients and their carers on a daily basis. Once the gatekeeper has judged a patient well enough to be approached, they will give the patient a ‘consent to contact’ card. If the patient and/or their carer is happy to discuss the study with a researcher, a member of the research team will go to the ward to provide the patient with a study information sheet. The researcher will answer any questions about the study and give the patient and carer time (as much as is needed) to consider participating. Then, when they are ready and if willing to participate, consent will be sought either before discharge, or if discharged, consent will be taken on the day of the interview.

The recruitment target is 5–10 patients with a hip fracture (and 5–10 of their carers), 5–10 readmitted patients (and 5–10 of their carers) and 5–10 healthcare staff from a variety of the following professional groups—medical, nursing, allied health professional and care home manager. This recruitment target is realistic over a 4-month period, when using gatekeepers who are not part of the dedicated research staff; we estimate that between 4 and 6 patients with hip fracture are admitted per day via the Emergency Department in the acute hospital site. The literature describes several system and patient-level factors that may influence 30-day readmission rate; therefore, the whole pathway will be analysed by targeting both admitted and readmitted patient groups. A 30-day readmission is an admission to hospital within 30 days of discharge from hospital after the index incident (the original admission due to hip fracture). Where participants have capacity but are unable to give written consent, verbal consent will be taken, and the researcher will sign the consent form on behalf of the participant in the presence of an independent witness.

A multiperspective approach will enable the research team to seek consent from the carers/family members of enrolled patients. Before patients give consent, they will understand that they will be asked to nominate a carer or family member and then consent would be obtained from each individual (patient and carer) in turn. This will allow data from patients to be linked with that of their carers or family members to explore how perspectives compare. If it is not possible to recruit the carer at the same time as the patient, the patient will be recruited. This is because understanding the perspective of patients who do not have an obvious carer or family member may also be important to understanding readmission. When interviewing a carer or family member separately from the patient, care will be taken to maintain the confidentiality of the patient, particularly as carers and family members may be concerned about what the patient has said.^22^

Each patient participant will be interviewed once. The researchers will aim to conduct the interview within 1 month of being enrolled. Interviews which are scheduled post discharge will take place in a variety of settings as some patients may be discharged into residential care and others to their own home (or equivalent domestic setting).

All interviews will be semistructured for 30–45 min. If English is not the patient’s first language, and they would like an interpreter, the research team will aim to provide one to facilitate participation. All interviews will be audio recorded, but where this is not practical or the interviewee refuses permission, field notes will be taken.

The semistructured interview topic guide was designed to provide a deeper understanding of the patient journey (including transitions between care locations and discharge planning—for example) and which interventions might (acts or omissions) mitigate readmission risk (see online supplemental file 2).

Interviews will be transcribed verbatim and coded manually (in Microsoft Word or Excel) or via NVivo analysis software. We will follow guidance from Braun and Clarke *et al.*^20 21^ Analysis will occur concurrently with data collection. In the next step, we will follow guidance from Vogl *et al,*^23 24^ where inter-related accounts from members of the same social unit (a patient and a carer) will be compared and triangulated.

### Data management and archiving

Confidentiality will be strictly maintained; participant data will be anonymised wherever possible.

All electronic study data will be stored in the Chief Investigator’s Higher Education Institute secure Research Data Store (RDS) which is password protected and only accessible to members of the ARTHUR study management group. Non-electronic data (eg, signed consent forms) will be kept securely at the recruiting site. All data will be stored for the duration of the study. The sponsor’s and NHS Trust’s current archiving procedures will be adhered to. Data will be stored according to guidance from the sponsor for a maximum of 10 years after the study closes.

### Safety reporting

Work Package 2 uses qualitative methodology, and it is unlikely that there would be any adverse reactions recorded during the observations or interview study. If there is any distress caused to the patient or their carer, it will be documented in the patients’ medical records. Where a participant reports or discloses safety concerns, or where the researchers are concerned for the patient’s welfare, or in the unlikely event a member of the study team witnesses a safety issue, the ARTHUR study team will follow site-specific standard operating procedures.

### Patient and public involvement statement

We also acknowledge our ARTHUR study Patient and Public Involvement group and members of the Clinical Research Ambassador Group (CRAG) for their advice and support during the construction of the research question and study procedures.

## Ethics and dissemination

This study will be conducted in accordance with the principles of the Declaration of Helsinki and in full conformity with relevant regulations and with the ICH Guidelines for Good Clinical Practice ICH GCH E6 R2 (2017).^25^ This research will also be governed by the UK policy framework for Health and Social Care research, which sets out the principles of good practice in the management and conduct of health and social care research. NHS research ethics approval has been obtained (REC 23/WM/0242). PIONEER have UK Health Research Authority and Research Ethical Committee approvals as a research database and can provide ethics under licence for this dataset.^18^ Ethical approvals for the PIONEER data are provided by the East Midlands – Derby REC (20/EM/0158).

### Discussion and limitations

There is currently a global call to action to provide better care for people who experience fragility fracture who are growing in volume along with the global ageing population.^26^ In England, while far less people die after a fragility fracture of the hip, the number of people who are readmitted within 30 days of discharge remains stubbornly high and this is especially problematic for people who experience health inequalities and/or living in deprivation. The ARTHUR study attempts to understand what avoidable readmission means in a large urban centre in the UK in order to design an intervention or bundle of interventions which could be trialled in the future. This is important so that the people who need to come back to hospital continue to be readmitted; whereas, those that do not have the right care in place to maximise their recovery outside of the acute hospital setting. Any intervention(s) developed to reduce readmission can successfully support those people who can safely stay at home to remain there.

Patient and Public Involvement (PPI) can help to improve the quality of research which aims to tackle health inequalities through improving study design, data collection and analysis.^27^ However, the ARTHUR team have identified early challenges to obtaining genuine representation of the regional community. The known trend of ‘recruiting the usual suspects’ in PPI is beginning to be reversed and researchers are being called to task to get out into the community to meet the diverse communities, take time to get to know them, build trust and break down any barriers that may contribute to making people feel alienated or afraid to contribute to a research group.^28^ Yet, the resource restrictions within academic research teams may still be prohibitive to this.

This study is not without risk and the contingencies to mitigate these have been carefully considered. First, research involving older adults, particularly who have had a sudden traumatic event like a hip fracture which may have resulted in a change of living arrangements such as move to a care home, can surface emotive concerns such as illness, vulnerability and end-of-life. Based on advice provided by the National Institute for Health and Care Research (NIHR) ENRICH (Enabling Research In Care Homes) group, the team will keep a reflexive diary and discuss any issues that are causing concern in the PPI meetings, study management group meetings and with their mentors. Second, given the stresses some patients experience after a traumatic injury, some of these experiences may be exposed in the interviews. The researcher is trained to help if this happens. The ARTHUR study research team will refer to a standard operating procedure on how to remedy distress during an interview if needed. Third, there is a risk of inadequate recruitment. This study aims to recruit patients who live in deprived and ethnically diverse areas which may be challenging. We will minimise the risk associated with recruitment bias by employing a translator where needed and by implementing strong PPI engagement. A time frame of 4 months has been planned to conduct WP2 which will allow adequate patient flow (based on existing throughput) to enable us to hit our recruitment targets. Finally, the risk that participants may be identified will be mitigated by adopting unique participant identification codes. Every effort will be made to ensure it is not possible to identify who took part in the study. The ARTHUR study team recognise the need for further intervention development work to be conducted with a patient population who experience cognitive impairment in the future. It is estimated that around one quarter of patients with hip fractures experience cognitive impairment,^29 30^ are at higher risk of surgical and medical complications and are often excluded from clinical research.^31^

Through the use of mixed methods, this study will explore ‘avoidable’ 30-day readmission after hip fracture and explain which interventions are most capable of reducing avoidable readmission after fragility fracture of the hip in the Midlands area of England. Future studies will (i) explore pertinent issues relating to readmission in persons with cognitive impairment and (ii) design and test interventions which may be capable of reducing avoidable readmission.

## Supplementary material

10.1136/bmjopen-2024-094163 online supplemental file 1

10.1136/bmjopen-2024-094163 online supplemental file 2

## Data Availability

Data sharing not applicable as no datasets generated and/or analysed for this study. Data are available upon reasonable request. Data may be obtained from a third party and are not publicly available. All data relevant to the study are included in the article or uploaded as supplementary information.
