# Supplementary material for: Understanding readmission after hip fracture: a mixed methods study protocol
Source: BMJ Open. 2025 Jul 15;15(7):e094163. doi: 10.1136/bmjopen-2024-094163 (PMC12265812; doi:10.1136/bmjopen-2024-094163)
Supplement: online supplemental file 1 [file bmjopen-15-7-s001.docx]

**ARTHUR - Avoiding re admission after hip fracture**

**Supplemental file 1: Observation guide**

This guide is to be used by the ARTHUR researcher as an aid to support writing field notes.

**Aim**

To describe system-based challenges affecting service delivery and discharge processes for hip fracture patients at [SITE]

We expect to collect data on:

- What counts as an avoidable readmission
- When readmission could have been avoided/in what circumstances
- Different staff perspectives on the same issue – how do people arrive at a decision/how are processes ‘enacted’ by staff in practice?

**Description of the site:**

Record a brief overview of the setting (number of beds, location, acute or community setting, accommodation, etc.)

**Procedural**

- Routines (handover, safety huddles, ward rounds, admission process)
- Workflow, busy periods, tensions
- Admission/referral process for patients –any discussion of related risk of readmission?
- Process of caring for patients who might not be recovering as they should. Who are staff worried about, how do they discuss these concerns and to who/when?
- Process of escalating deteriorating patients - who is involved how, who does what, what happens next, what information/support do staff draw on why

**Relational:**

- Professional power / hierarchies
- Socialisation / identity / belonging
- Team composition and roles
- Supervision and support

**Patient and family role:**

- Involvement in care, sharing of concerns
- Formal versus informal mechanisms of involvement
- Tensions / constraints

**Rescue events or incidents**

- Significant or important events / incidents
- Contextual influencing factors
- Variations and exceptions to any emerging patterns

**In each area visited explore:**

**Pre-existing risk factors for re-admission risk**

Notice discussions between staff members on risk of readmission, including ASA grade, BMI, age, length of stay, co-morbidities

**The MDT:**

How are decisions made about who is reviewed and who is not reviewed

Who are the specialists involved in the patients care? Who is involved in the regular review of the patient?

Orthogeriatric roles?

Specialist Trauma Nurses?

Discharge team?

Are junior doctors part of the team reviewing the patient? Are they comfortable in seeking senior support? Likewise Advanced Practitioners/Physician Associates?

Social worker?

**Staff comments on access to services (in acute trust or community):**

Pharmacy and medications review/reconciliation

Physiotherapy

Occupational Therapy

Speech and Language Therapy

Chaplaincy

Social work

Diagnostic services – e.g labs for blood/path results, imaging

**Transition of care**

Between ED and the ward (observed conversations, ED will not be a setting for observation)

Between the ward and the discharge lounge

Between the discharge lounge and home

**Preparedness for discharge and involvement in discharge planning**

Patients and family, care givers and friends – involvement, concerns expressed (observed conversations of staff members, the ward/bays will not be a setting for observation)

Documentation of plans/checklists

**Post discharge support**

Care package plans

Telephone follow up

GP appointments
