# Supplementary material for: Understanding readmission after hip fracture: a mixed methods study protocol
Source: BMJ Open. 2025 Jul 15;15(7):e094163. doi: 10.1136/bmjopen-2024-094163 (PMC12265812; doi:10.1136/bmjopen-2024-094163)
Supplement: online supplemental file 2 [file bmjopen-15-7-s002.docx]

**Title: ARTHUR - Avoiding readmission after hip fracture**

**Supplemental File 2: Interview topic guide**

Semi structured schedule – can be adapted for patients/carers and staff

This guide is to be used by the ARTHUR researcher as an aid to support the interview conduct

The following questions need not be covered in this particular order, but rather, the interview should flow freely and as naturally as possible. The interviewer will prompt with phrases such as ‘can you tell me a little more about that’ can you give me an example’

| Opener | Thank you for participating in this study. My name is [XX]. I am a [INSERT ROLE] within [INSERT SITE]. I will be asking questions for about 30 minutes, but we’ll try to keep it shorter than this if we can. I will ask you about what you think about your care after your experience of hip fracture in particular relating to your discharge from hospital. I value your views.  Please speak as freely as you wish. For the purposes of the interview I will not refer to you by name and we will try not to identify anyone by name, but if you do mention someone’s name we will anonymise the written transcript so they cannot be identified. Please be assured that all your responses will be anonymised and you will not be able to be identified by any written material relating to this interview. You are free to stop the interview at any time |
| --- | --- |
| Before we start | - Have you read the information sheet? - Is it OK if we record the interview? - Please can I check for your consent for your transcript to be reviewed by any member of our ARTHUR study team? - Have you got any questions? |
| *Tell me about you* | Health state, brief history (inc coming into hospital) and current situation (including housing/place of residence) |
| *On being in hospital:* | Example prompts/topics  How has it been / how was it in hospital?  Is there anything you have felt worried or unhappy about?  Did the doctor see you?  Do you know which specialty staff were from and whether they are a consultant, junior, etc.?  How much did staff talked to you about what’s going on?  Did you have enough chance to ask questions?  How quickly have things been sorted out for you, and have there been any delays or problems?  How secure and confident have you felt/did you feel during your stay? |
| *On preparing for discharge* | Do you understand what will happen in terms of your recovery - timeline, further appointments, what support you might need?  Did you have any specific concerns about leaving hospital?  How confident were you that you would manage when you left hospital?  Will you contact other organisations for support (use appropriate examples – e.g Age UK? The Royal Voluntary Service? Military Veterans?)  What do you think you may have needed to make sure you feel confident to be discharged? What would help you most? |
| Any significant events during healthcare episode | Significant or important events / incidents (from the perspective of the participant) |
| Transition of care | - Between ED and the ward - Between the ward and the discharge lounge - Between the discharge lounge and home/discharge setting |
| Patient and family role | - Involvement in care, sharing of concerns - Formal versus informal mechanisms of involvement - Tensions / constraints |
| Post discharge support | Care package plans  Telephone follow up  GP appointments  *On managing complications*  If something went wrong when you are at home do you think you would seek help?  How confident would you feel about seeking help from your GP if you felt unwell or weren’t sure about something like medication?  *On compliance with treatment/advice*  What treatment/advice was received on preparing for discharge/post discharge?  What was helpful/unhelpful? |
| Depravation domains and health inequalities  (e.g. housing quality). | e.g - are they in the private rented sector (PRS), social rented sector (SRS) or a home owner, the state of repair of the property, and the impact of housing on their health and well being. |
| Communication | Language/understanding/hearing – were their needs accommodated for? |
| Ask specifically about risk of re admission | What do you think may contribute towards patients returning to hospital within a month of going home?  Why might this happen? What should be done to prevent it? |
| EXIT QUESTIONS | Is there anything else you want to tell me?  Thank you for your participation |
