# Supplementary material for: Understanding readmission after hip fracture: a mixed methods study protocol
Source: BMJ Open. 2025 Jul 15;15(7):e094163. doi: 10.1136/bmjopen-2024-094163 (PMC12265812; doi:10.1136/bmjopen-2024-094163)
Supplement: Supplementary file 3 [file bmjopen-2024-094163.reviewer_comments.pdf]

PEER REVIEW HISTORY

BMJ Open publishes all reviews undertaken for accepted manuscripts. Reviewers are asked to complete a checklist review form and are provided with free text boxes to elaborate on their assessment. These free text comments are reproduced below.

ARTICLE DETAILS

Title (Provisional)

Understanding re admission after hip fracture: a mixed methods study protocol

Authors

Sutton, Emma; Rahman, Usama; Reilly, Hannah; Miller, Caroline; Vedutla, Tarunya; Melody, Teresa; Gudivada, Ranaganayaki; Topping, AE

VERSION 1 - REVIEW

**Reviewer**	**1**
**Name**	**Kenanidis, Eusthathios**
**Affiliation**	**Aristotle University of Thessaloniki**
**Date**	**02-Nov-2024**
**COI**	**None**

This study protocol presents a compelling approach to understanding the key factors that contribute to readmission following a hip fracture. By identifying these reasons, we can enhance patient care and reduce return rates, ultimately benefiting both patients and healthcare systems. The researchers are dedicated to uncovering the key factors contributing to 30-day readmissions following hip fracture surgery; by employing a combination of quantitative methods—such as health data analysis—and qualitative approaches, including in-depth interviews and observations, they seek to provide a comprehensive understanding of this critical issue. This study will take place in a large, ethnically diverse city, offering rich insights; however, these findings may not represent the broader population effectively. The study team acknowledges several challenges, but the protocol thoroughly explores the key difficulties and limitations of this research, providing a transparent understanding of its scope. I currently have no other comments to make.

**Reviewer**	**2**
**Name**	**Reider, Lisa**
**Affiliation**	**Johns Hopkins University**
**Date**	**14-Nov-2024**

COI **None**

This protocol paper describes a mixed methods study designed to better understand avoidable 30-day readmissions following hospitalization for hip fracture in older patients to inform the development of interventions geared toward readmission prevention. The study targets two hospitals in the UK Midlands region that treat patients with predominantly low socioeconomic status, a risk factor for readmission. The study has 4 parts: W1 is a retrospective analysis of hospital data to determine prevalence of 30 day readmission and to identify factors associated with readmission; W2 is a qualitative study of patients, caregivers and health care staff to learn about care pathways and experiences that may contribute to avoidable readmission; W3 is a workshop of clinicians and patients and their care givers to review the findings of the study and possible solutions; and W4 is to design an intervention to reduce readmissions and implementation plan. A major strength of the study is a focus on underserved populations who are at highest risk of poor outcomes and who may benefit most from targeted intervention. Although interesting, the paper lacks considerable detail regarding the methods and analysis.

1. The title should include the study type, per journal instructions: i.e. "a mixed methods study"

2. According to the journal instructions, the last section of the structured abstract should be about ethics and dissemination.

3. A goal of this study is to define avoidable hospital readmission, however it is unclear from the methods how this will be done. Is there an existing framework or baseline definition for avoidable readmission as a starting point? How will the quantitative and qualitative analyses confirm or expand our current understanding of avoidable readmission?

4. W1- What are the years of the five-year period for data analysis? The authors say that the data will be used to identify trends, but are these annual trends? How will "incident" hip fracture be defined for purposes of identifying 30-day readmission? How is deprivation defined? How is reason for readmission defined (e.g., admitting diagnosis)? Will the analysis be primarily descriptive, or will statistics be used to evaluate trends and associations?

5. W2- What does non participant and MT mean?

6. W2- how are patients, caregivers and staff identified for interviews? Is there consideration given to SES status in the sampling since that seems to be an important factor highlighted in this study? What is the justification for "5-10"? Should the inclusion criteria be restricted to those who have had a 30-day readmission since the goal is to learn about avoidable readmissions? Can the authors provide example questions or topics, factors, or themes that will be explored during the interviews?

7. W3- It sounds like the goal of the workshop is to get feedback on the results of the quantitative and qualitative analysis and on proposed interventions; however, no detail is

given regarding what the interventions are or how they will be selected. There is also no description of how feedback will be collected and organized.

VERSION 1 - AUTHOR RESPONSE

Reviewer 1

Reviewer comment	action
This study protocol presents a compelling approach to understanding the key factors that contribute to readmission following a hip fracture. By identifying these reasons, we can enhance patient care and reduce return rates, ultimately benefiting both patients and healthcare systems. The researchers are dedicated to uncovering the key factors contributing to 30-day readmissions following hip fracture surgery; by employing a combination of quantitative methods—such as health data analysis—and qualitative approaches, including in-depth interviews and observations, they seek to provide a comprehensive understanding of this critical issue. This study will take place in a large, ethnically diverse city, offering rich insights; however, these findings may not represent the broader population effectively. The study team acknowledges several challenges, but the protocol thoroughly explores the key difficulties and limitations of this research, providing a transparent understanding of its scope. I currently have no other comments to make.	None taken<br><br>Many thanks for your thoughtful comments

Reviewer 2

1. The title should include the study type, per journal instructions: i.e. "a mixed methods study"	We have changed the title - Avoiding re admission after hip fracture study: study protocol for a mixed methods intervention development study
2. According to the journal instructions, the last section of the structured abstract should be about ethics and dissemination.	Section added: **Ethics and dissemination** This protocol has regard for the HRA guidance and was submitted via IRAS: 330074 and obtained UK NHS REC approval: 23/WM/0242.
3. A goal of this study is to define avoidable hospital readmission, however it is unclear from the methods how this will be done. Is there an existing framework or baseline definition for avoidable readmission as a starting point? How will the quantitative and qualitative analyses confirm or expand our current understanding of avoidable readmission?	We have re-phrased the aim because it is answered primarily by the qualitative study - how is avoidable readmission defined and categorised by caregivers and patients?<br>We have clarified the introduction: it is not yet known which specific combination of interventions are effective to reduce readmissions which could be avoidable[17]<br><br>and we have clarified the methods:<br>Using mixed methods this study will:

	• Explore how avoidable readmission is discussed by caregivers and patients readmission<br><br>We have also removed phrasing relating to 'defining' avoidable' replacing with 'explore'<br><br>We think the text in the introduction shows that while the literature does not provide clarity on exactly which factors constitute avoidable, a picture is building on which factors may *inffuence* 30-day readmission, and we think this is clear in the introductory text - Some of these factors may be amenable to change and may therefore, may create a picture of in which circumstances 30-day readmission might be avoided. For example, a recent observational study showed that hospitals with more staff grades and/or junior doctors preoperative fluid management protocols in place; data recorded for the time between discharge and initiation of community therapy; and plans in place to reconfigure hip fracture services - were associated with fewer 30-day readmissions.<br><br>The ARTHUR study is exploratory, and will use qualitative methods to explore what avoidable means with the study participants.
4. W1- What are the years of the five-year period for data analysis? The authors say that the data will be used to identify trends, but are these annual trends? How will "incident" hip fracture be defined for purposes of identifying 30-day readmission? How is deprivation defined? How is reason for readmission defined (e.g., admitting diagnosis)? Will the analysis be primarily descriptive, or will statistics be used to evaluate trends and associations?	Brackets have been added to specify the dates - Five years of anonymised data on admitted fragility hip fracture patients from an acute NHS Trust in the Midlands region of England,(01/01/2019 - 31/12/2023) will be retrospectively analysed.<br><br>A section with regards to planned analyses has been added to W1 identifying how incident hip fractures will be obtained, as well as readmission analyses.
5. W2- What does non participant and MT mean?	A definition of non-participant has been added - Non-participant observations (where researchers collect data without actually interacting or participating in the activity being observed)<br><br>Apologies, I could not find the term 'MD'
6. W2- how are patients, caregivers and staff identified for interviews? Is there consideration given to SES status in the	A section on identification has been added:

sampling since that seems to be an important factor highlighted in this study? What is the justification for "5-10"? Should the inclusion criteria be restricted to those who have had a 30-day readmission since the goal is to learn about avoidable readmissions? Can the authors provide example questions or topics, factors, or themes that will be explored during the interviews?	***Identification of patients and carers:*** The gatekeepers for the interview study will be part of the patient's healthcare team and they are able to identify patients who are eligible for the interview study. A member of the ARTHUR study team will telephone (or visit) the 'gatekeeper' to identify patients with a hip fracture on a daily basis. Once the gatekeeper has deemed the patient well enough to be approached, the gatekeeper will give the patient a 'consent to contact' card. If the patient is happy to discuss the study with the researcher, a member of the research team will go to the ward to provide the patient with a study information sheet. The researcher will answer any questions about the study and give the patient time (as much as is needed) to consider participating. Then, when they are ready and if willing to participate, consent will be sought either before discharge, or consent can be taken on the day of the interview.<br><br>***SES*** – in this study it was not possible to ask the gatekeepers to screen for SES, the criteria for this is not routinely collected or used by the admitting teams/healthcare staff<br><br>***Recruitment target – sentence added:*** This recruitment target is realistic over a four month period, when utilising gatekeepers who are not dedicated research staff because we estimate that between 4 and 6 patients with hip fracture are admitted per day via the Emergency Department in the acute hospital site.<br><br>**Should the inclusion criteria be restricted to those who have had a 30-day readmission** – sentence added to justify our approach: The literature describes several system and patient level factors that may influence 30-day readmission rate, therefore, the whole pathway will be analysed by targeting both admitted and re-admitted patient groups.<br><br>**Can the authors provide example questions or topics, factors, or themes that will be explored during the interviews?** – sentence added below and topic guide added as an appendix<br>The semi-structured interview topic guide was designed to provide a deeper understanding of

	the patient journey (including transitions between care locations and discharge planning – for example) and which interventions might (acts or omissions) mitigate readmission risk (see appendix 1)
7. W3- It sounds like the goal of the workshop is to get feedback on the results of the quantitative and qualitative analysis and on proposed interventions; however, no detail is given regarding what the interventions are or how they will be selected. There is also no description of how feedback will be collected and organized.	Work package 3 and 4 have been removed and will be reported in a future protocol when the intervention is developed and tested. We have added a paragraph to this effect in the discussion
